# Variation of Human Immunodeficiency Virus Type-1 Reverse Transcriptase within the Simian Immunodeficiency Virus Genome of RT-SHIV

**DOI:** 10.1371/journal.pone.0086997

**Published:** 2014-01-31

**Authors:** Debra A. Wadford, Robert C. Kauffman, Jesse D. Deere, Scott T. Aoki, Richard A. Stanton, Joanne Higgins, Koen K. A. Van Rompay, Andradi Villalobos, James H. Nettles, Raymond F. Schinazi, Niels C. Pedersen, Thomas W. North

**Affiliations:** 1 Center for Comparative Medicine, University of California Davis, Davis, California, United States of America; 2 Children's Center for Drug Discovery (CDD), Departments of Pediatrics and Biomedical Informatics, Emory University School of Medicine, Atlanta, Georgia, United States of America; 3 California National Primate Research Center, University of California Davis, Davis, California, United States of America; 4 Emory University School of Medicine, Veterans Affairs Medical Center, Decatur, Georgia, United States of America; 5 Department of Medicine and Epidemiology, School of Veterinary Medicine, University of California Davis, Davis, California, United States of America; 6 Department of Molecular Biosciences, School of Veterinary Medicine, University of California Davis, Davis, California, United States of America; Institut National de la Santé et de la Recherche Médicale, France

## Abstract

RT-SHIV is a chimera of simian immunodeficiency virus (SIV) containing the reverse transcriptase (RT)-encoding region of human immunodeficiency virus type 1 (HIV-1) within the backbone of SIV_mac239_. It has been used in a non-human primate model for studies of non-nucleoside RT inhibitors (NNRTI) and highly active antiretroviral therapy (HAART). We and others have identified several mutations that arise in the "foreign" HIV-1 RT of RT-SHIV during *in vivo* replication. In this study we catalogued amino acid substitutions in the HIV-1 RT and in regions of the SIV backbone with which RT interacts that emerged 30 weeks post-infection from seven RT-SHIV-infected rhesus macaques. The virus set points varied from relatively high virus load, moderate virus load, to undetectable virus load. The G196R substitution in RT was detected from 6 of 7 animals at week 4 post-infection and remained in virus from 4 of 6 animals at week 30. Virus from four high virus load animals showed several common mutations within RT, including L74V or V75L, G196R, L214F, and K275R. The foreign RT from high virus load isolates exhibited as much variation as that of the highly variable envelope surface glycoprotein, and 10-fold higher than that of the native RT of SIV_mac239_. Isolates from moderate virus load animals showed much less variation in the foreign RT than the high virus load isolates. No variation was found in SIV_mac239_ genes known to interact with RT. Our results demonstrate substantial adaptation of the foreign HIV-1 RT in RT-SHIV-infected macaques, which most likely reflects selective pressure upon the foreign RT to attain optimal activity within the context of the chimeric RT-SHIV and the rhesus macaque host.

## Introduction

Human immunodeficiency virus type 1 (HIV-1) and simian immunodeficiency virus (SIV_mac239_) are two closely related lentiviruses that induce a similar progressive persistent infection and acquired immunodeficiency syndrome (AIDS) in humans and rhesus macaques respectively [1]. Although the reverse transcriptase (RT) of SIV_mac239_ shares 60% sequence similarity with the RT of HIV-1 [2] it is not susceptible to the non-nucleoside RT inhibitors (NNRTI) used in AIDS therapy [3]. To develop an animal model for the study of HIV-1 therapy using this class of RT inhibitors, Uberla et al. engineered a chimeric virus containing the RT of HIV-1 HXBc2 within the backbone of SIV_mac239_, designated RT-SHIV [4]. Despite having a foreign RT, the chimeric RT-SHIV replicates well in rhesus macaques, recapitulates SIV_mac239_ in its pathology [4]–[6], and has proven to be an important model of HIV-1 therapy [7], [8]. The RT-SHIV/rhesus macaque model also provides a unique opportunity to examine adaptation of a foreign enzyme (HIV-1 RT) in the context of a closely related genome (SIV_mac239_). Considering that RT-SHIV replicates in macaques to levels similar to HIV-1 in infected humans [4]–[7], [9]–[13], then the number of replication cycles of RT-SHIV over a given period of time should be comparable to that of HIV-1. Thus, we expect every possible mutation to arise numerous times per day, as described by Coffin for HIV-1 [13]. Given that RT-SHIV is not a naturally occurring virus, some of these mutations may result in variants with enhanced *in vivo* fitness, relative to the original RT-SHIV inoculum, that will therefore dominate the virus population.

The RTs of HIV-1 and SIV each reverse transcribes viral RNA into DNA utilizing the host’s cellular tRNA^Lys^ as a primer to initiate RNA-dependent-DNA polymerization [14], [15]. The RT enzymes of both HIV-1 and SIV lack true editing capability resulting in an error prone reverse transcription of the RNA genome. Reverse transcription consists of several coordinated steps whereby RT interacts not only with the host’s tRNA^Lys^ [16]–[20], but also with several other components including the viral RNA template, dNTPs, the *cis*-acting transactivation response element (TAR) stem-loop in the 5′ untranslated region (5′ UTR) of viral RNA [21], [22], the nucleocapsid (NC) protein [23]–[28], and the Tat protein [29], [30]. Considering these multiple interactions of RT, suboptimal replication efficiency and/or processivity of the foreign RT within RT-SHIV could result in decreased viral fitness. Indeed, *in vitro* replication of the original RT-SHIV construct was shown to be severely impaired and was rescued by the emergence of a single point mutation in the primer binding site (PBS) located in the 5′ UTR of RT-SHIV [15]. This single point mutation restores the PBS to the cognate PBS of HIV-1 [31]–[33], resulting in a dramatic increase in the replication of RT-SHIV in the human T-B lymphoblast cell line CEMx174 [15]. Interestingly, few other point mutations are detected in the RT of RT-SHIV when cultured in CEMx174 cells [15]. However, *in vivo* studies have shown that RT-SHIV isolates from rhesus macaques did acquire point mutations in the RT-encoding region [4]–[6], [34].

Here we report on variation arising within the foreign RT-encoding region of *pol* and in several domains known to interact with RT including the 5′ UTR, NC, and Tat protein of RT-SHIV isolated from seven infected rhesus macaques. We compared the difference in variation between the putatively more conserved RT-encoding region with that of the highly variable surface glycoprotein (gp120), which has been extensively characterized in SIV from infected macaques [35]–[38]. As a comparator of RT variation from RT-SHIV infected macaques we also assessed the variation of the cognate RT of SIV_mac239_ isolated from infected rhesus macaques.

## Materials and Methods

### Ethics Statement

All samples used in this study were from a previously published and approved animal study [7] with no additional animals used. This study was approved by the Association for the Assessment and Accreditation of Laboratory Animal Care, International (AAALAC) accredited University of California, Davis Institutional Care and Use Committee (IACUC). The UC Davis IACUC has an Animal Welfare Assurance on file with the Office of Laboratory Animal Welfare (OLAW). Animals were administered 10 mg/kg body weight ketamine-HCl (Parke-Davis, Morris Plains, NJ, USA) intramuscularly when necessary for immobilization. Additionally, analgesics were administered at the discretion of the California National Primate Research Center (CNPRC) veterinary staff in an effort to minimize all animal pain and discomfort. Macaques were housed at the CNPRC, which is fully accredited by the Association for the Assessment and Accreditation of Laboratory Animal Care (AAALAC). For housing, animals were maintained in cages with 4 square feet of floor space, or 6 square feet if over 10 kg, and fixed perch bars in a temperature-controlled BSL-2+ vivarium with continuous monitoring of temperature and humidity. Compatible animals were paired continuously or intermittently (separated at night) whenever possible. All animals had visual and auditory access to other macaques 24 hours per day. These animals were fed a balanced commercial macaque chow (Purina Mills, Gray Summit, MO) twice daily and fresh produce twice weekly, with free access to water 24 hours per day. Supplemental food was provided when clinically indicated. Environmental enrichment was provided daily, included manipulanda (forage boards, mirrors, puzzle feeders) and novel foodstuffs. The seven animals described in this study were used as controls in the previously reported study [7] of highly active antiretroviral therapy (HAART) in RT-SHIV-infected rhesus macaques with the endpoint of the study determined by experimental design. All macaques were humanely euthanized by overdose of sodium (60 mg/kg) pentobarbital administered by the intravenous route under ketamine sedation (10 mg/kg).

### Animals, Virus Inoculation, and Blood Collection

Seven juvenile rhesus macaques (*Macaca mulatta*) 7 to 10 months old (∼1.3 to 2 kg) from the retrovirus-free colony of the CNPRC were each inoculated intravenously with 1.0 ml of cell-free virus containing 10^5^ 50% tissue culture infectious doses (TCID_50_) of RT-SHIV, as previously described [7]. EDTA-anticoagulated blood samples were collected regularly to obtain plasma and peripheral blood mononuclear cells (PBMC). Plasma was also the source of viral RNA. When necessary, animals were immobilized with ketamine-HCl (Parke-Davis, Morris Plaines, NJ), 10 mg/kg body weight, injected intramuscularly.

### Virus and Cells

Infectious RT-SHIV stocks were prepared as previously described [7]. RT-SHIV used in this study contained the T to C substitution at position 8 of the SIV tRNA primer binding site, which is necessary for rapid replication of RT-SHIV [15]. CEM×174 cells, which are permissive for both HIV-1 and SIV [39], were grown as previously described [7].

### Serial Passage of RT-SHIV in CEMx174 and Rhesus PBMC

For the first round of serial passages of RT-SHIV (WT or variants) in CEM×174 cells, triplicate, cultures containing 0.5 to 1×10^6^ cells/ml were infected at an input multiplicity of infection (MOI) of 0.001. The second and third rounds were performed by adding 50–200 µl of the tissue culture fluid (TCF) from the previous round to CEMx174 cell suspensions containing 0.5 to 1×10^6^ cells/ml.

PBMC for serial passages of RT-SHIV (WT/196R) were isolated from donor monkeys and grown in RPMI 1640 supplemented with 20% heat-inactivated fetal bovine serum (FBS), 200 U/ml recombinant human IL-2, 100 U/ml penicillin, 0.1 mg/ml streptomycin, and 2.0 mM L-glutamine (PBMC complete medium). Prior to infection, rhesus PBMC cultures were stimulated with 0.5 µg/ml staphylococcal enterotoxin A (SEA) (Sigma-Aldrich, St. Louis, MO) for 60–72 hours. Five rounds of serial passage of wild-type RT-SHIV in rhesus PBMC were performed in triplicate in three separate experiments. For the first passage of each experiment, 0.5 to 2×10^6^ SEA stimulated PBMC were infected at a MOI of 0.001. Each subsequent passage was initiated by adding 50–200 µl of cell free TCF obtained from the previous round to fresh uninfected PBMC. All cultures were maintained in PBMC complete medium at 37°C in a humidified 5% CO_2_ atmosphere. Passages were regularly monitored by SIV p27 ELISA and the medium was changed twice a week by replacing one-quarter to one-half of the total volume with fresh growth medium. The first experiment utilized PBMC from two macaques (25042 and 28034). These cells were obtained fresh before each round of passage. After being separately stimulated with SEA, the cells were mixed 1∶1 immediately prior to infection. Experiments two and three used PBMC from either macaque 32397 or 32319 respectively.

To evaluate stability of the G196R mutation, RT-SHIV-196R was serially passaged in rhesus PBMC. During this experiment the first round of passage was performed in PBMC from macaque 28717 and the second through fourth rounds of passage were performed in cells from macaque 32397.

For each serial passage experiment, infected cells and TCF were isolated by centrifugation at 500×g two times at the completion of each round. Proviral DNA was extracted from cell pellets using the Qiagen DNAeasy kit according to the manufacturer’s instructions (Qiagen, Valencia, CA). Isolated proviral DNA was then amplified by nested PCR. First round PCR used primers 239-2571 and 239-4751(R); second round PCR used primers 239-2794 and 239-4673(R) (Table S1). PCR products were directly sequenced using primers 239-2841, HXB2-3018, and HXB2-3509 (Table S1).

### Virus Isolation from Infected Macaques

RT-SHIV from weeks 4 and 30 post-inoculation was isolated from PBMC or plasma of infected rhesus macaques by co-culture with CEMx174 cells in 25-cm^2^ flasks and monitored for viral replication by SIV p27 core antigen as described by Lohman et al. [40]. Virus-positive cultures were centrifuged twice at 600×*g* for 5 min to remove cells and supernatants were stored at −80°C. Titers of virus stocks were determined by the focal infectivity assay as described by Murry et al. [41], and these stocks were used for studies of replication kinetics. Cells were resuspended in phosphate-buffered saline and stored at −80°C for subsequent DNA extraction, PCR, and sequence analysis of proviral DNA.

### Determination of Viral RNA Levels in Plasma and Cell Culture Supernatants

A real-time quantitative RT-PCR (TaqMan) assay with a sensitivity of 50 copies of viral RNA/ml plasma or cell culture supernatant was used to quantify RT-SHIV RNA as previously described [42].

### Nucleic Acid Preparation and Sequence Analysis

To characterize the genetic landscape of the RT-SHIV inoculum, we performed next generation sequence analysis using a 454 sequencer on selected regions of the RT-SHIV inoculum. These regions encoded RT amino acids 41 to 296 and envelope-gp120 amino acids 1 to 526. Detailed methods describing RNA extraction, amplicon generation, sequencing, and data analyses are described in supplementary materials (Text S1: 454 Inoculum Sequencing).

Proviral DNA from PBMC or plasma co-cultures and DNA from RT-PCR amplification of plasma viral RNA were examined by sequence analysis. Nucleotides of SIV are numbered beginning from the 5′-end of the SIV genome (i.e., position 1 of SIV corresponds to nucleotide 257 of the GenBank reference M33262 proviral sequence). Five regions of the genome were examined: (*i*) the 5′ UTR from nucleotides (nt) 518 to 1052 (in SIV_mac239_); (*ii*) the nucleocapsid-encoding region from nt 2018 to 2565; (*iii*) the reverse transcriptase-encoding region from nt 2852 to 4531 (in RT-SHIV); (*iv*) the envelope (*env*) gene from nt 6607 to 9246 (in SIV_mac239_); and (*v*) *tat* exon 1 from nt 6558 to 6853 and *tat* exon 2 from nt 9062 to 9158 (in SIV_mac239_). Total cellular DNA from infected CEMx174 cells was extracted using a DNeasy Tissue Kit (Qiagen, Valencia, CA) following the manufacturer’s protocols. Aliquots of 2–10 µl of each DNA preparation were amplified by nested PCR using JumpStart REDTaq (Sigma-Aldrich, St. Louis, MO) and 0.4 µM of each primer to generate DNA fragments for sequence analysis. All primers and primer sequences are listed in Table S1. PCR products were purified using a commercial PCR Purification Kit (Qiagen). DNA sequence analyses were performed as previously described [41], [43] with 3 µM of sequencing primers. The RT region of week 30 high VL plasma viral RNA and isolates from PBMC and plasma was independently amplified five times by PCR and each resulting DNA mixture was sequenced to determine reproducibility of the method. Each independent analysis yielded identical results.

The 5′ UTR was amplified in the first round using primers 239-44 and 239-1807(R) and in the second round with primers 239-290 and 239-1474(R). The 5′ UTR sequencing primers were 239-384 and 239-1142(R). The NC-encoding region was amplified in the first round with primers 239-1779 and HXB2-3341(R) and in the second round with primers 239-1860 and 239-2591(R). Sequencing primers for NC were 239-2018 and 239-2565(R). The RT-encoding region was amplified in the first round with primers 239-2571 and 239-4751(R) and in the second round with primers 239-2675 and 239-4615(R). The RT-encoding region was sequenced using primers 239-2786, HXB2-3145, and HXB2-3837. DNA sequence analyses of the RT-encoding region of SIV_mac239_ were performed as previously described [41].

The *env* gene was amplified in three sections also employing nested PCR. The first fragment of *env* was amplified using first round PCR primers 239-6353 and 239-7697(R) and second round PCR primers 239-6463 and 239-7593(R). The second *env* fragment was amplified using first round primers 239-7016 and 239-9064(R) and second round primers 239-7589 and 239-8969(R). The final *env* fragment was amplified with first round primers 239-8075 and 239-9878(R) and second round primers 239-8961 and 239-9735(R). The three resulting *env* fragments were sequenced using the following primers: 239-6463, 239-7016, 239-7589, 239-8075, 239-8961, 239-7593(R), 239-8969(R), 239-9266(R), and 239-9703(R). Exon 1 of *tat* was amplified in the first round with primers 239-4682 and 239-7697(R) and in the second round with primers 239-5811 and 239-7593 (R). Exon 1 of *tat* was sequenced with primers 239-5811 and 239-6474 (R), while *env* sequence from primer 239-8969(R) was used to determine the sequence of *tat* exon 2.

Plasma viral RNA was reverse transcribed and amplified by PCR to generate DNA for sequence analysis of the RT-encoding region. Viral RNA was extracted from 140–560 µl of cell-free plasma using a commercial viral RNA extraction kit (Qiagen) as per manufacturer’s instructions. Synthesis of cDNA and the first round of PCR were carried out using a one-step RT-PCR mix (Invitrogen) according to the manufacturer’s recommended conditions with 5–20 µl of viral RNA and 2 µM each of the primers 239-2571 and 239-4751(R) or 239-2675 and HXB2-3253(R), as previously described [41]. Second round fragments were amplified with primers 239-2786 and HXB2-3253(R) or 239-2675 and 239-4615(R) and JumpStart RED Taq polymerase (Sigma-Aldrich), as previously described [7].

**Figure 1:**
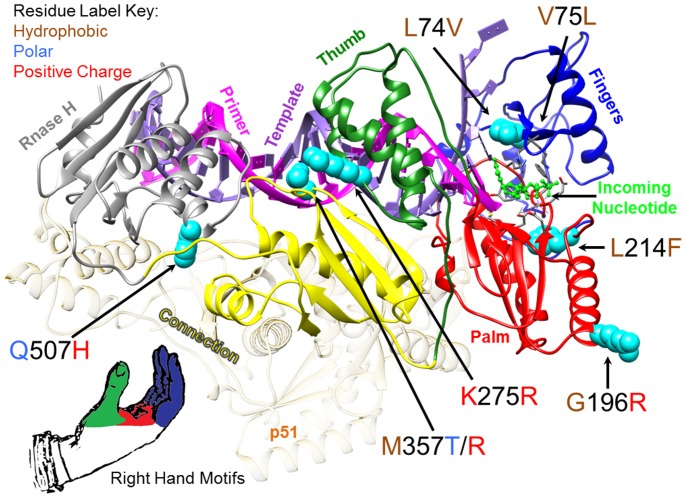
HIV-1 Reverse Transcriptase (RT) showing amino acid substitutions detected in RT-SHIV high virus load rhesus macaques. HIV-1 RT p66 subunit ribbon diagram depicted with the most frequently detected amino acid substitutions as cyan-colored spheres. The domains of the RT active site are colored to correspond to the model of RT analogous to a human right hand with the fingers domain as dark blue, the palm domain as red, and the thumb domain as green. The X-ray crystal structure PDB ID: 1RTD [45] of pre-catalytic, wild type HIV-1 reverse transcriptase in complex with double stranded DNA and incoming nucleotide was used to make the image.

Figure 1 shows the locations of RT mutations that were frequently observed *in vivo.* In order to generate Figure 1, the X-ray crystal structure coordinates for a wild type HIV-1 RT ternary complex containing DNA primer/template and an incoming nucleotide were downloaded from the Protein Data Bank (PDB) at www.pdb.org [44] (PDB ID:1RTD [45]). RT mutations were mapped onto both the p66 and p51 subunits and analyzed for potential structure/function relations using UCSF Chimera [46]. The most relevant positions, located on the p66 subunit, were assessed relative to structural motifs as defined by Kohlstaedt et al. [47]. The Figure 1 image was produced using UCSF Chimera [46].

### Determination of MHC Class I Mamu-A*01 Allele Status and CD4^+^/CD8^+^ Ratios

Total cellular DNA was extracted from PBMC of each animal using a DNeasy Tissue Kit (Qiagen) as described above. The presence of the MHC class I Mamu-A*01 allele was determined by a PCR-based method described by Knapp et al. [48]. CD4^+^/CD8^+^ T cell ratios were calculated from percentages of CD4^+^ and CD8^+^ T cells as determined by analysis of blood lymphocytes with a FACS Calibur flow cytometer (Becton Dickinson, Franklin Lakes, NJ) as previously described [7].

### Calculation of Synonymous and Nonsynonymous Mutation Frequencies

Mutation frequencies were calculated by dividing the number of nucleotide substitutions (point mutations) by the total number of nucleotides in the coding region, 1575 and 1680 nucleotides for SIV_mac239_ gp120 and HIV-1 RT, respectively. The coding region of SIV_mac239_ RT contains 1677 nucleotides. Average mutation frequencies of RT or gp120 were calculated by determining the arithmetic mean of the sum of synonymous and nonsynonymous mutation frequencies. Some of the RT-SHIV isolates contained deleted regions within gp120. For these isolates, gp120 mutation frequencies were calculated in two ways, by including and excluding deletions. When deletions were included in the calculation, one deleted nucleotide was considered to be one mutation.

### Determination of Viral Replication Capacity

Replication experiments were conducted using virus isolated from PBMC co-cultures. For each isolate, at least three independent experiments were performed, each in triplicate. Viral RNA in culture supernatants was quantified by real-time RT-PCR and these values were used to assess relative replication capacities. CEMx174 cells (10^6^ cells per culture) were infected with virus at an MOI of 0.01 in a volume of 0.5–1.0 ml RPMI 1640 (Invitrogen) supplemented with 0.1% FBS at 37°C in a humidified 5% CO_2_ atmosphere. After 2.0–2.5 hours of adsorption, cells were washed with RPMI 1640 supplemented with 0.1% FBS and centrifuged at 500×*g* for 5 min three times to remove unadsorbed virus. Infected cells were then resuspended in 7 ml of RPMI 1640 supplemented with 10% heat-inactivated FBS, 100 U/ml penicillin, 0.1 mg/ml streptomycin, and 2.0 mM L-glutamine (complete RPMI 1640 medium) and incubated for 7 days at 37°C in a humidified 5% CO_2_ atmosphere. Every 1–2 days, after cells were allowed to settle at the bottom of each flask, 2 ml aliquots of cell culture fluid were removed and centrifuged for 2 min at 9,000×*g* to pellet any remaining cells. Supernatants were stored at –80°C for subsequent viral RNA extraction and quantification, as described above. Fresh complete RPMI 1640 medium was added to each culture to restore the volume to 7 ml. Replication curves represent the average of three replicates with error bars representing standard error of the mean for that experiment. Relative replication capacities were evaluated for statistical significance at 5 days post-infection using Welch's t test when comparing isolates from animals to the RT-SHIV control. Statistical significance comparing replication capacities of same-animal isolates was evaluated using paired two-sample t-tests. P values less than 0.05 were considered statistically significant.

### Site Directed Construction of Reverse Transcriptase Mutants

Infectious RT-SHIVs containing G196R, K275R, and both G196R and K275R, mutations in reverse transcriptase (RT) were produced by site-directed PCR mutagenesis of the RT-SHIV 5′-half clone. The mutagenized 5′-half clones were then used to produce infectious virus as previously described [7]. Construction of the three mutagenized RT-SHIV 5′-half clones was accomplished by overlap extension PCR mutagenesis. Primers for these reactions are shown in Table S2. PCR mutagenesis was carried out in two stages. In the first stage two separate reactions were performed: one PCR from a flanking upstream positive sense primer, HXB2–2681, to the negative-sense mutagenesis primer and a second PCR from a positive-sense mutagenesis primer to a flanking downstream antisense primer 239-5294(R). First stage PCR utilized the wild type 5′-RT-SHIV half clone as the DNA template for construction of both the G196R mutant and the K275R mutant; the mutagenesis primers were G196R:mutF/mutR and K275R:mutF/mutR respectively. First stage PCR for the construction of the G196R and K275R double mutant was performed using the completed K275R construct as the DNA template and the G196R-mutF/mutR mutagenesis primers. The second stage of the mutagenesis utilized first stage PCR products as the DNA template for a PCR using the flanking primers HXB2–2681 and 239-5294(R). PCR products were cloned by TOPO-TA cloning according to manufacturer’s instructions (Invitrogen, Carlsbad, CA). Finally, the desired mutant 5′-RT-SHIV half clone constructs were produced by subcloning the mutagenized PCR inserts into the wild-type 5′-RT-SHIV half clone using unique *EcoRV* and *PacI* restriction sites that flank RT amino acid positions G196 and K275 respectively.

In order to limit the amount of time required post-transfection to produce infectious virus, the 5′-RT-SHIV half clone was constructed to contain a single nucleotide substitution of thymine to cytosine at position eight of the SIV primer binding as described by Soderberg, et al. [15].

## Results

### Mutations in the RT-SHIV Inoculum

As originally described by Soderberg et al., the thymine to cytosine (TC) nucleotide substitution at position 8 (nucleotide 829) in the primer binding site (PBS) was present in the inoculating stock of RT-SHIV [15]. In order to assess whether or not other mutations detected *in vivo* were a component of the original RT-SHIV inoculum, we sequenced the nucleotide regions that encoded RT amino acids 41 to 296 and envelope-gp120 amino acids 1 to 526. These regions were sequenced using a 454 sequencer at an approximately 2,000X sequencing depth. The 454 sequence analysis identified a few nonsynonymous mutations that were greater than the pre-defined 0.5% sequence read threshold. Mutations observed within RT were G196R (5.0%) and E204K (1.74%). Mutations observed within envelope-gp120 were N146K (0.73%) and G347R (0.73%).

### Virus Isolates from RT-SHIV Infected Animals

**Figure 2:**
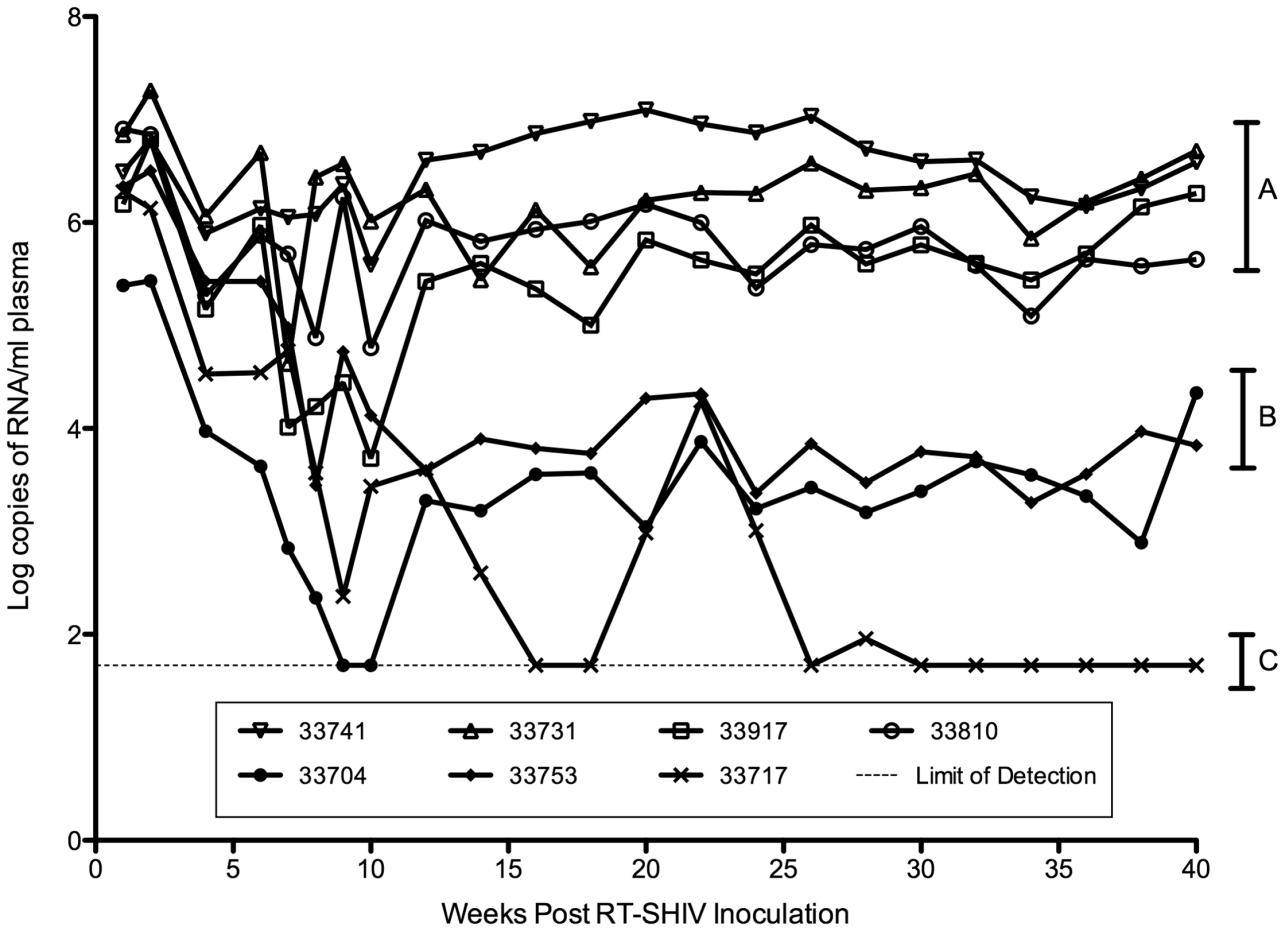
Plasma viral RNA levels in RT-SHIV-infected rhesus macaques. By week 30 post-inoculation, virus loads had segregated the animals into 3 groups: A) animals with high virus loads (0.6–3.9×10^6^ RNA copies/ml plasma); B) animals with moderate virus loads (2.5–6.0×10^3^); and C) one animal with a virus load less than 50 RNA copies/ml plasma which is below the limit of detection (dashed line). The data displayed in this figure were obtained from North et al [7].

RT-SHIV isolates and plasma viral RNA were from seven infected rhesus macaques that were used as untreated controls in a study of HAART by North et al. [7]. These isolates were obtained from animals at 4 and 30 weeks post-inoculation (PI). Information for each animal including virus load, gender, and Mamu A *01 allele haplotypes is shown in Table 1. At 4 weeks PI virus loads ranged from 9.4×10^3^ to 1.2×10^6^ RNA copies/ml of plasma (Table 1). By 30 weeks PI, virus set points segregated the animals into one of three distinct groups: four animals had virus loads (VLs) ranging from 0.6–3.9×10^6^ RNA copies/ml plasma (“high VL” animals) (Figure 2, group A); two animals had VLs of 2.5×10^3^ and 6.0×10^3^ RNA copies/ml plasma (“moderate VL” animals) (Figure 2, group B); and one animal had a VL below the limit of detection of 50 RNA copies/ml plasma (Figure 2, group C).

**Table 1:** Characteristics of RT-SHIV-infected rhesus macaques examined in this study.

		Virus load (×10^3^)^a^			CD4:CD8		
Animal	Gender	Wk 4	Wk 30	Relative virus load	Wk 4	Wk 30	Mamu A*01 Haplotype
33717	Female	38	–b	–b	1.98	1.93	Homozygous
33704	Female	9	3	M^c^	2.58	2.38	Absent
33753	Female	270	6	M	2.19	2.55	Absent
33731	Male	1,200	2,200	H^d^	1.04	0.75	Heterozygous
33741	Female	780	3,900	H	1.78	1.58	Absent
33810	Female	200	920	H	1.83	1.22	Heterozygous
33917	Female	140	610	H	0.84	1.01	Absent

a RNA copies/ml plasma.

b Undetectable (<50 RNA copies/ml plasma).

c Moderate virus load.

d High virus load.

The presence of the major histocompatibility complex (MHC) class I allele Mamu-A*01 has been associated with control of virus replication in SIV_mac239_-infected rhesus macaques [49], [50]. Three of the seven animals in this study were Mamu-A*01 positive (Table 1). Two of the Mamu-A*01 positive animals (33741 and 33810) had high VLs at week 30, while the third Mamu-A*01 positive animal (33717) had no detectable VL at week 30 (Table 1). Both moderate VL animals (33704 and 33753) and the two remaining high VL animals (33741 and 33917) were Mamu-A*01 negative (Table 1). Our results suggest that, for RT-SHIV-infected animals, homozygosity at Mamu-A*01 correlated with control of virus replication while heterozygosity at this allele did not (Table 1).

### Mutations in RT-SHIV Isolates

Mutations in the HIV-1 RT-encoding region of RT-SHIV and in regions of the SIV_mac239_ backbone (5′ UTR, NC, and *tat* exons 1 and 2) were identified by Sanger sequencing. Variation in *env*, especially within gp120, was used as a standard of reference for a highly variable region [51], and variation of the SIV_mac239_ RT-encoding region was used as a control for variability of RT.

Mutations were present in RT-SHIV by 4 weeks PI (Table 2). Sequence analyses of plasma viral RNA revealed that virus from 6 of the 7 animals contained the G196R substitution in RT. G196R was present in infectious RT-SHIV isolated from PBMC co-cultivations from 3 of these animals and 2 of the 7 animals had virus with K275R in RT (Table 2). RT-SHIV from animal 33810 at week 4 had no mutations in RT (Table 2).

**Table 2:** Amino acid substitutions detected in the RT-encoding region of RT-SHIV isolated from rhesus macaques at 4 and 30 weeks post-inoculation.

	Week 4		Week 30			
Animal	Plasma^a^	PBMC^b^	Plasma^a^	PBMC^b^	Plasma^c^	Virus load^d^
33717	G196R	G196R	e	f	f	Undetectable
	K275R	K275R				
33704	G196R	T69N	– g	–	f	Moderate
		E79K				
		G196R				
33753	G196R	G196R	G196R	G196R	f	Moderate
			Q507H			
33731	G196G/R	–	V75L	V75L	V75L	High
			G196R	G196R	G196R	
			L214F	L214F	L214F	
			K275R	K275R	K275R	
			M357T	M357T	M357T	
			Q507H	Q507H	Q507H	
33741	G196R	K275R	V75L	L74V	V75L	High
			G196R	G196R	G196R	
			L214F	L214F	L214F	
			K275R	K275R	K275R	
					K558R	
33810	–	–	V75L	V75L	V75L	High
			L214F	L214F	L214F	
			Q507H	M357R	M357R	
				Q507H	Q507H	
				H208L	R277K	
					T286A	
33917	G196R	–	L74V	L74V	L74V	High
			K103N	K103N	K103N	
			G196R	G196R	G196R	
			P225H	P225H	P225H	
					V108I	
					E203K	

a Plasma viral RNA.

b PBMC co-culture proviral DNA.

c Plasma co-culture proviral DNA.

d Relative virus load at week 30.

e No detectable viral RNA.

f No virus isolated.

g Wild-type virus.

By 16 weeks PI, VL set points had been established in the 7 animals as shown in Figure 2. Animals with high VLs showed an overall increase in the frequency of RT mutations from week 4 to week 30 relative to animals with moderate VLs (Table 2). At week 30, the G196R RT mutation was present in RT-SHIV from 4 of the 6 animals with detectable virus load (Table 2). High VL animals had RT-SHIV with the following RT mutations: either L74V or V75L, which resulted in RT with tandemly repeated leucine or valine at residues 74 and 75 (4 of 4 animals); G196R (3 of 4 animals); L214F (3 of 4 animals); K275R (2 of 4 animals); M357T (1 of 4 animals) or M357R (1 of 4 animals); and Q507H (2 of 4 animals).

*Env* variation in SIV_mac239_ has been associated with changes in cellular tropism as well as adaptation to and escape from host immune selective pressures [38], [52], [53]. Sequence analyses of the entire open reading frame of *env* at week 4 revealed 1 or 2 mutations in virus from 5 of 7 animals; 3 isolates had R751G (Table 3). As shown in Table 3, infectious virus from animals with detectable VL at 30 weeks contained several more amino acid substitutions in Env than observed at week 4. Although deletions were detected within gp120 in some isolates, no deletions were observed in RT. RT-SHIV from 3 high VL animals (33917, 33741, and 33810) contained a higher frequency of amino acid substitutions in gp120 than RT-SHIV from moderate VL animals, but isolates from moderate VL animals had more substitutions in gp41 than high VL animals (Table 3). Many of the observed Env substitutions listed in Table 3 have been reported by others to occur in SIV_mac239_-infected animals [35], [37], [38], [52]–[56].

**Table 3:** Amino acid substitutions detected in Env (gp120 and gp41) of RT-SHIV isolated from rhesus macaques at 4 and 30 weeks post-inoculation.

	Week 4	Week 30				
	Envelope	gp120		gp41		
Animal	PBMC^a^	PBMC^a^	Plasma^b^	PBMC^a^	Plasma^b^	Virus load^c^
33717	N405K	d	d	d	d	Undetectable
33704	*–* e	V67M R435K	d	R751G L802F R813QV837A L852I	d	Moderate
33753	R751G Q835R	I40V R120K T136A A417V	d	P741L R751G R813QA848V	d	Moderate
33731	*–*	V67L D511G	V67L D511G K125ET136M Δ421–426	R751G V837G	I702V R751G V837G	High
33741	R751G	L4P V67M Q164H A417T P421S D511G	V67M S116N A138TK141E Q164H T173SK355R A417T P421S	S627K R751G	S627K R751G	High
33810	L24Q	G62D V67M W345R P421T D511G Δ415–420	G62D V67M S135PW345R G347E D511NΔ415–421	V562L R751G	M543I V562L R751G	High
33917	R751G	V67M N523S Δ415–420^f^	V67M D511G N523SΔ135–137 Δ423–426	R751G	K631E R751G	High

a PBMC co-culture proviral DNA.

b Plasma co-culture proviral DNA.

c Relative virus load at week 30.

d No virus isolated from plasma at week 30.

e Wild-type virus.

f “Δ” notation represents a deletion at the specified residues.

In addition to assessing variation of the foreign RT relative to the cognate Env of RT-SHIV, we investigated whether certain regions of SIV_mac239_ may have acquired mutations indicative of adaptation to the introduced HIV-1 RT. Sequence analyses of virus isolates were performed in the regions encoding viral components known to interact with RT such as the 5′ UTR, NC, and Tat. The thymine to cytosine (T→C) nucleotide substitution at position 8 (nucleotide 829) in the primer binding site (PBS) was present in the inoculating stock of RT-SHIV and was maintained in all isolates at week 4 and week 30 (data not shown). No other substitutions were detected at week 4 in the 5′ UTR (data not shown). A maximum of three additional nucleotide substitutions were detected in the 5′ UTR at week 30 as shown in Table 4. These substitutions were detected in either the TAR stem-loop (from nucleotides 519 to 643) or the PBS stem-loop (from nucleotides 717 to 898 in the 5′ UTR). No substitutions were observed in the 5′ UTR downstream of the PBS stem-loop (nucleotides 899 to 1052).

**Table 4:** Nucleotide substitutions^a^ detected in the 5′ untranslated region at 30 weeks post-inoculation.

Animal	PBMC^b^	Plasma^c^	Virus load^d^
33717	e	e	Undetectable
33704	C566T G728A G775A^f^	e	Moderate
33753	A519G A602G A803T	e	Moderate
33731	–	C641T	High
33741	C641C/T G893A	C541T G893A	High
33810	C745T	G589A C745T	High
33917	–	G635A G741A	High

a The T829C substitution (in the 8th position of the PBS- see text) is not shown because it was present in the RT-SHIV inoculating stock and was maintained in all isolates examined.

b PBMC co-culture proviral DNA.

c Plasma co-culture proviral DNA.

d Relative virus load at week 30.

e No virus isolated.

f Numbers represent nucleotide positions starting from the 5′-end of the SIV RNA genome (i.e. position 1 of SIV corresponds to nucleotide 257 of the GenBank reference M33262 proviral sequence).

Mutations were not detected in the NC-encoding region of virus from any of the 7 animals at week 4 (data not shown). At week 30, RT-SHIV from two animals (33741 and 33753) had one nonsynonymous (NS) mutation each in NC: RT-SHIV from high VL animal 33741 had the A431V substitution (amino acids numbered from Gag start site); virus from moderate VL animal 33753 contained a mixture of both wild-type and P390L in the NC (data not shown). Week 4 RT-SHIV isolates contained no mutations in exon 2 of *tat* (data not shown); *tat* exon 1 was not examined at week 4. Few substitutions were observed in Tat at week 30: two high VL isolates (33731 and 33810) had the S82P substitution in Tat; the two other high VL isolates contained the A121E (33741) or R106Q (33917) Tat substitutions; moderate VL isolate 33753 had two substitutions in Tat: E24K and A25V (data not shown).

### Frequency of Nonsynonymous RT Mutations

To quantify the variation detected in RT and gp120 of RT-SHIV isolates we calculated the frequency of both synonymous and nonsynonymous (NS) mutations within each coding region at week 30. RT-SHIV isolates from high VL animals demonstrated higher total frequencies of RT point mutations (synonymous plus NS mutations) than isolates from animals with a moderate VL (Table 5). Comparisons between NS mutations in RT and gp120 of isolates from high VL animals revealed that the typically conserved RT showed no significant difference in variation relative to the highly variable gp120 (Table 6).

**Table 5:** Total mutation frequencies.

			Total Mutation Frequency (x10^−3^)^a^			
Virus	Isolation Method	Animal	RT	gp120 Without gaps^b^	Gp120 With gaps^c^	Relative virus load
RT-SHIV^d^	Plasma co-culture	33731	5	3	7	High
		33741	4	8	8	High
		33810	5	6	10	High
		33917	5	5	10	High
RT-SHIV^d^	PBMC co-culture	33731	5	1	1	High
		33741	4	4	4	High
		33810	4	4	8	High
		33917	4	2	6	High
		33704	1	2	2	Moderate
		33753	2	3	3	Moderate
SIVmac239^e^	PBMC co-culture	31304	2	ND	ND	Moderate
		31339	1	ND	ND	High
		31632	1	ND	ND	High

a Total mutation frequency is expressed as the number of substitutions per 1000 nucleotides and defined as the sum of the frequencies of synonymous mutations plus non-synonymous mutations.

b Nucleotide deletions (gaps) were excluded in calculations of mutation frequency.

c Nucleotide deletions (gaps) were included in calculations of mutation frequency.

d Isolated from rhesus macaques at 30 weeks post-inoculation.

e Isolated from rhesus macaques at 40 weeks post-inoculation.

ND: Not determined.

**Table 6:** Average frequency of nonsynonymous mutations.

Virus load category	Virus	RT^a^	gp120a,b
High virus load	RT-SHIV^c^	3×10^−3^	3×10^−3^
	SIVmac239^d^	3×10^−4^	ND
Moderate virus load	RT-SHIV^e^	3×10^−4^	2×10^−3^
	SIVmac239 f	0	ND

a Average Frequency of NS Mutations is expressed as substitutions per nucleotide.

b Nucleotide deletions were excluded in calculations of mutation frequency.

c Isolated from four RT-SHIV-infected rhesus macaques at 30 weeks post-inoculation (PI).

d Isolated from two SIVmac239-infected rhesus macaques at 40 weeks PI [43].

e Isolated from two RT-SHIV-infected rhesus macaques at 30 weeks PI.

f Isolated from one SIVmac239-infected rhesus macaque at 40 weeks PI [43].

ND: Not determined.

To ascertain the difference in variation of the foreign RT within RT-SHIV relative to that of the cognate RT of SIV_mac239_, we calculated the frequency of synonymous and NS mutations that emerged in the RT of SIV_mac239_ collected from three animals at 40 weeks PI. These SIV_mac239_-infected animals were used in a previous study conducted by our laboratory [43]. One SIV_mac239_-infected animal (31304) had a moderate VL of 5×10^3^ RNA copies/ml plasma; the other two animals had high VLs: animal 31339 had a VL of 6×10^5^ RNA copies/ml plasma, while animal 31632 had a VL of 1×10^6^ RNA copies/ml plasma [43]. Remarkably, the RT-encoding regions of viral isolates from RT-SHIV-infected animals with high VLs had a 10-fold higher average frequency of NS mutations relative to isolates from SIV_mac239_-infected animals with high VLs (*p*<0.025; Table 6).

### *In vitro* Replication Capacity of RT-SHIV Isolates

**Figure 3:**
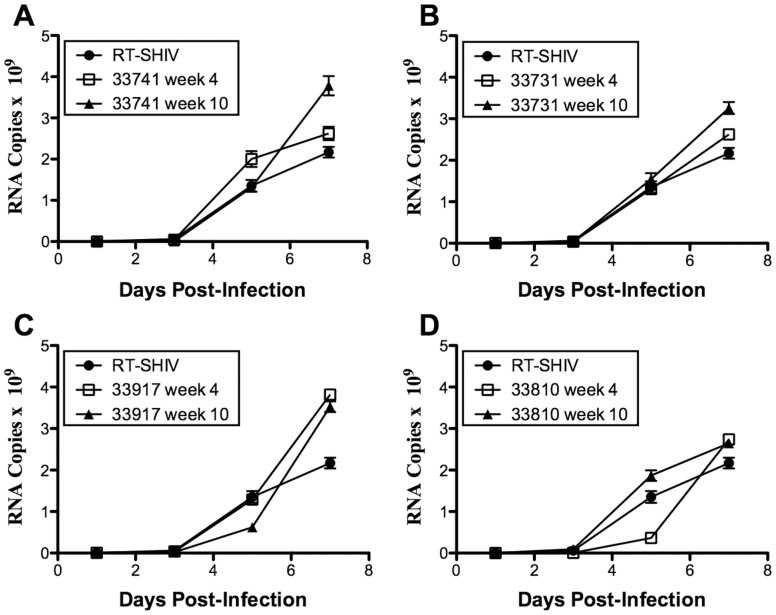
Replication kinetics of RT-SHIV isolated from rhesus macaques. CEMx174 cells were infected with RT-SHIV isolates from animals or the inoculating RT-SHIV stock as a control (see Materials and Methods). Replication was evaluated by measuring viral RNA copies per ml of culture supernatant for 7 days following infection. For each isolate, three independent experiments were performed in triplicate. Each experiment showed similar replication trends for the isolates relative to each other and the RT-SHIV control, therefore only representative curves are shown. Error bars indicate standard error of the mean for that experiment. RT-SHIV isolates from (A) animal 33741; (B) animal 33917; (C) animal 33731; (D) animal 33810.

Replication kinetics of RT-SHIV isolates in CEMx174 cells did not correlate with VLs in animals at week 30 vs. week 4. Plasma viral RNA levels increased 1.8-fold to 5-fold in high VL animals from week 4 to week 30 (Table 1), yet RT-SHIV isolated from only one of four animals with high VL (33810) had higher *in vitro* replication capacity at week 30 relative to its week 4 counterpart (Figure 3D; *p*≤0.03). For two animals with high VL (33741 and 33917), the week 4 isolates had higher *in vitro* replication capacities than the week 30 isolates (Figure 3A, *p*≤0.006 and Fig. 2B, *p*≤0.03, respectively). There was no difference in replication capacities of isolates from week 4 and 30 of animal 33731 (Figure 3C). None of the isolates from the high VL animals exhibited significantly different *in vitro* replication capacities relative to the RT-SHIV control (Figure 3).

### Selection of G196R Variants of RT-SHIV in Rhesus PBMC

Serial passages of RT-SHIV in PBMC cultures from uninfected rhesus macaques were performed to determine whether any of the RT mutations observed *in vivo* would emerge *in vitro*. These passages were performed in triplicate in mixed lymphocyte cultures containing stimulated PBMCs from two different macaques (donors #1 and #2). Proviral DNA was sequenced after each passage. The G196R mutation emerged in RT in all replicates, became the predominant sequence by the second passage, and was maintained through subsequent passages (Table 7). In one culture another RT mutation, H208L, transiently appeared during the first passage, but was not present in subsequent passages. None of the other *in vivo* RT mutations emerged *in vitro*.

**Table 7:** Reverse transcriptase codon 196 in RT-SHIV isolates from serial passage in cultured cells.

		Cell type used				
Passage	Replicate	CEMx174	Mixed PBMCDonors 1&2	Non-mixedPBMC Donor 3	Non-mixedPBMC Donor 4	Non-mixedPBMC Donor 3
Zero^a^		G	G	G	G	R
One	A	G	G	G^b^	G	R
	B	G	G	G	G	R
	C	G	G	G	G	R
Two	A	G	R	G^b^	G	R
	B	G	R	G	G	R
	C	G	R	G	G	R
Three	A	G	R	ND	G	R
	B	G	R	ND	G	R
	C	G	R	ND	G	R
Four	A	G	R	G & R	ND	R
	B	G	R	G	G	R
	C	G	R	G	G	R
Five	A	G	R	G & R	G	
	B	G	R	G	G	
	C	G	R	G & R	ND	

a Input Virus.

b A minor peak of the G196R mutant sequence was detectable, but the predominant sequence was wild-type.

ND: Not determined.

Interestingly, selection for the G196R mutation was not as strong in PBMC cultures from individual donor macaques. In PBMC from one macaque (donor #3) the population was a mixture of G and R at codon 196 after passage 4 of culture A and passage 5 of both cultures A and C (Table 7). The G196R mutation was not detected in any of the replicates during 5 rounds of serial passage of RT-SHIV in PBMC from another macaque (donor #4) (Table 7).

We also evaluated replication fitness of a site-directed G196R reverse transcriptase mutant of RT-SHIV in non-mixed cultures of rhesus macaque PBMCs. Replication kinetics of this mutant was not significantly different from wild-type RT-SHIV (data not shown). Moreover, there was no detectable reversion of this mutant upon serial passage in rhesus PBMCs (Table 7).

Serial passage of RT-SHIV in the human CEMx174 cell line did not result in the emergence of the G196R or K275R variants after five rounds of passage (data not shown). We have also never detected these mutations in any of the RT-SHIV stocks we have grown in this cell line using conventional bulk sequencing. However, the G196R mutation was detected as a minor component (5.0%) of the RT-SHIV inoculum following 454 deep sequencing (1,900X sequencing depth). In addition, there was no reversion upon three rounds of serial passage of the RT-SHIV mutants (G196R, K275R or the G196R, K275R double mutant) in CEMx174 (data not shown). Thus there was neither selection for nor against these mutations in CEMx174 cells.

## Discussion

Regions of lentivirus genomes that encode viral proteins susceptible to host immune responses show extensive variability [35], [36], [38], [54], [57]–[60]. In particular, the surface glycoproteins of lentiviruses are highly variable [61], whereas other lentiviral proteins, such as the capsid and RT of HIV-1, are much less variable [62]. Results of this study showed that the foreign RT of RT-SHIV exhibited a level of variation similar to that of the highly variable gp120 surface glycoprotein and 10-fold higher than that of the cognate RT of SIV. This atypical variation of HIV-1 RT is most likely a reflection of the selective pressure on the foreign RT to attain optimal activity and processivity within the context of the chimeric RT-SHIV and the rhesus macaque host. This hypothesis is supported from the virus load data (Figure 2) and RT substitution frequency data presented in Table 5. As noted, two macaques had virus loads that were 2–3 orders of magnitude lower than those of the four high VL macaques at week 30 (Table 1). The observed differences in virus loads suggest that the number of productively infected cells and viral burst size were much less in moderate VL animals than in high VL animals, as indicated by the robust CD4:CD8 ratios observed in the 2 moderate VL animals at both weeks 4 and 30 post-inoculation (Table 1). Nevertheless, the variation of gp120 in moderate VL isolates was similar to that of high VL isolates (Table 6). However, RT variation in moderate VL isolates was much less than that exhibited by high VL isolates. Thus, it is apparent that the selective pressures driving variation of the HIV-1 RT in RT-SHIV are different from the pressures that drive selection of envelope variants. The extent of gp120 variation despite reduced virus loads in moderate VL animals suggests that the immune responses of these animals are controlling RT-SHIV infection better than in high VL animals. It is possible that the RT-SHIV isolates from moderate VL animals have lower *in vivo* replication fitness than isolates from high VL animals, or that variations in host genetic background or antiviral immune responses play a role.

RT-SHIV isolates from high VL animals exhibited amino acid substitutions in all domains of RT including the fingers (L74V and V75L), the palm (G196R, L214F), the thumb (K275R), and the connection (M357 R or T), as well as in RNase H (Q507H). Some of these substitutions have been previously reported in other studies of RT-SHIV isolated from infected rhesus macaques. Balzarini et al. observed the RT mutations L74V, K275R, M357T, and Q507H, but they did not report the appearance of G196R [5]. Hofman et al. identified the mutations L74V or V75L (tandemly repeated leucines or valines at residues 74 and 75), G196R, L214F, K275R, and M357T [6], [34]. The RT-SHIV inoculum was propagated in CEMx174 cells [7]. To characterize the genetic landscape of the RT-SHIV inoculum, we performed next generation sequence analysis using a 454 sequencer for selected regions of the RT-SHIV inoculum including the region encoding RT amino acids 41 to 296. Of the nonsynonymous mutations we detected in this study, only the RT-G196R mutation was detected at a relatively high level in the original inoculum (present in 5% of the 1899 sequence reads, data not shown). The G196R substitution arises from a G to A transition mutation at the first position of a GGG glycine codon, which is an APOBEC3G mutation site characterized by the GRD sequence motif [63]. It is unclear whether APOBEC3G activity during RT-SHIV replication in CEMx174 cells contributed to the 5% accumulation of the G196R substitution present in the RT-SHIV inoculum, but this substitution was not selected for in CEMx174-passaged RT-SHIV (Table 7). The 5% level of G196R present in the inoculum stock was not detectable by conventional bulk Sanger sequencing (data not shown).

Ambrose et al. have developed a similar RT-SHIV using the backbone genome of SIV_mne_ [64]. They tracked and documented the emergence of RT inhibitor resistance mutations from RT-SHIV_mne_-infected pig-tailed macaques under short course monotherapy with efavirenz and combination therapy with an NNRTI and two nucleoside RT inhibitors [65]. Similar to our findings, Ambrose et al. reported the consistent emergence of the V75L RT mutation [66] in the RT-SHIV_mne_. However in contrast to our study of untreated RT-SHIV-infected rhesus macaques, Ambrose et al. documented the prevalence of the V75L RT mutation in treated animals. These researchers did not specifically report the detection of G196R in their studies [67] whereas we consistently detected G196R and the tandem repeats of V or L at residues 74 and 75 in high virus load untreated animals. The differences in observed RT mutations between the RT-SHIV and RT-SHIV_mne_ models may reflect species-specific selection processes.

The G196 residue rarely mutates in HIV-1 and the G196R substitution in RT is rare or non-existent in HIV-1 [51], [68]. G196R was introduced into a cloned HIV-1 RT and was shown to have only 28% of the enzymatic activity of wild-type HIV-1 RT [69]. Despite its rarity and decreased enzymatic activity, the G196R substitution was detected in RT-SHIV isolates from 6 of 7 animals at week 4 and was present in virus from 4 of 6 animals with detectable virus load at week 30. G196R was also detected in RT-SHIV from 6 of 6 animals in two previous studies of RT-SHIV-infected rhesus macaques [6], [7]. The G196R substitution represents a major amino acid modification from a small neutral amino acid (glycine) to a large basic amino acid (arginine). The G196R mutation in RT did not emerge upon passage of RT-SHIV in human CEMx174 cells. However, it was strongly selected in RT-SHIV-infected macaques and by serial passage in mixed PBMC cultures containing PBMC from two macaques. In cultures from individual macaques, G196R was only weakly selected in PBMC from one macaque, and did not emerge in PBMC from the other macaque through five serial passages. The higher activation status of T lymphocytes of mixed PBMC cultures and of infected animals may account for the putative positive selection of G196R that we observed (Table 7).

The G196 residue of HIV-1 RT is located at the underside of the palm domain at an exposed surface of the enzyme [70] at the N-terminus of α-helix F of the p66-chain (Figure 1) [69]. Due to the exposed nature of the G196 residue and the prevalence of G196R detected from animal isolates, we hypothesize that a host factor may interact with RT at this residue and drive the selection for the G196R mutation in the foreign RT of RT-SHIV. Indeed, when an engineered G196R RT mutant was passed in rhesus PBMC, this mutation was stably maintained *in vitro* for the duration of four passages (Table 7). Mutation L214F, located just beyond the C-terminus of α-helix F is beneath the active site and may provide compensatory packing for host factor induced positioning of the helix. (Figure 1).

Despite the absence of drug therapy in the study animals, some of the RT-SHIV isolates demonstrated the emergence of mutations in RT known to confer drug resistance in HIV-1, such as L74V and K103N. Balzarini et al. [5] and Hofman et al. [6] have also reported the emergence of L74V and K103N, respectively, in RT-SHIV isolated from drug-naive rhesus macaques. The appearance of drug-resistance mutations has also been observed in HIV-1 infected humans not treated with antiretroviral therapy [71]. Presumably, these mutations had either a positive or neutral effect on RT-SHIV replication. Indeed, the K103N RT mutation in HIV-1 has little effect on viral fitness [72] and persists once established [73], [74]. Despite the emergence of K103N in those experiments, we did not detect this mutation in rebound viremia, upon cessation of therapy, in three experiments with RT-SHIV-infected macaques treated with an efavirenz-containing HAART regimen (efavirenz+PMPA+(−)-FTC or 3TC) [7], [8], [75]. Although the L74V mutation in RT reduced the *in vitro* replication fitness of HIV-1 [76], this mutation does not affect the processivity of the RT enzyme [77]. The significance of tandemly repeated leucine-leucine or valine-valine at residues 74 and 75 in the RT from all four animals with high virus loads (Table 2) is unclear, but the tandem leucine repeat was also documented in the RT-SHIV_mne_ model [66] which suggests a host-specific role driving the positive selection of these mutations. Their location within the finger domain involving incoming nucleotide and template positioning may offset a host-specific shifting of α-helix F.

The consistent emergence of RT mutations L74V/V75L, G196R, and L214F in RT-SHIV from 3 or more of the 4 high VL animals indicates that these mutations were positively selected for in virus from these animals. The significance of these RT mutations may involve increased processivity of the enzyme in the context of the foreign SIV_mac239_ backbone and the rhesus macaque host, as described above. These RT mutations may also play an as yet unrecognized role in immune/CTL escape in rhesus macaques. Because the MHC system in macaques is different from humans, it is not possible to say at this time whether human CTL epitopes correspond to macaque CTL epitopes. However a search of the literature did not reveal evidence that the RT substitutions we detected are associated with CTL escape mutations in HIV-infected humans. Future antiretroviral drug studies in rhesus macaques warrant testing RT-SHIV containing substitutions in RT such as V75L, G196R and L214F.

Mutations were not detected in SIV *cis*- or trans-acting factors known to interact with RT. This may be due to co-evolutionary dependency within the SIV_mac239_ genome imposed by packaging constraints or due to established interactions between 5′ UTR elements with both NC and Tat. Some variation was observed in these regions, but mutation frequencies were low and no single mutation was detected in more than two isolates. Interestingly, the few mutations detected within the 5′ UTR were located only in the TAR or PBS stem-loops, two regions with which RT is known to interact [21], [22], [78]. The lack of variation downstream of the PBS stem-loop is indicative of the highly conserved nature of this region which forms essential viral RNA secondary structures that are critical for virus assembly and packaging, such as the dimerization initiation site stem-loop and the encapsidation stem-loop [79]–[81].

## Conclusions

This study demonstrates that *in vivo,* specific mutations emerge in the chimeric RT-SHIV, regardless of drug therapy. The demonstrated atypical variation of the foreign RT of RT-SHIV in high virus load rhesus macaques, coupled with the positive selection of certain RT mutations, indicates that the HIV-1 RT evolved within the backbone of SIV_mac239_. We propose that these mutations confer an *in vivo* selective advantage to the chimeric RT-SHIV. The discordance between *in vivo* fitness and *in vitro* replication capacity underscores the importance and relevance of *in vivo* models like the rhesus macaque/RT-SHIV model for studies of viral fitness and resistance to AIDS therapies.

## Supporting Information

Table S1 **Primers used for PCR and DNA sequence analyses.**
(DOCX)

Table S2 **Primer Sequences for the Construction of site-directed RT mutants.**
(DOCX)

Text S1 **454 Inoculum Sequencing.**
(DOCX)
