# Supplementary material for: Variation of Human Immunodeficiency Virus Type-1 Reverse Transcriptase within the Simian Immunodeficiency Virus Genome of RT-SHIV
Source: PLoS One. 2014 Jan 31;9(1):e86997. doi: 10.1371/journal.pone.0086997 (PMC3909041; doi:10.1371/journal.pone.0086997)
Supplement: Table S1 — Primers used for PCR and DNA sequence analyses. (DOCX) [file pone.0086997.s001.docx]

**Table S1 - Primers used for PCR and DNA sequence analyses**

| Region*^a^* | Primer | 3' Base | Primer Sequence (5' → 3') |
| --- | --- | --- | --- |
| U5L | 239-44*^b^* | 44 | GCAAGAAGACATAGAATCTTAGAC |
|  | 239-290 | 290 | GGCCTACACTTATGAGGCA |
|  | 239-384 | 384 | CAAGAGGCCTTCTTAACATGG |
|  | 239-1142(R) | 1142 | CCCATACTACATGCTTCAAC |
|  | 239-1474(R) | 1474 | CAGGTGGACATAGTTACCAC |
|  | 239-1807(R) | 1807 | GGTATGGGGTTCTGTTGTC |
| NC | 239-1779 | 1779 | TATTGCAGGAACAACTAGTTCAG |
|  | 239-1860 | 1860 | CAGGAGATGGATCCAACTGG |
|  | 239-2018 | 2018 | AGAATTGGATGACTCAAACACTG |
|  | 239-2565(R) | 2565 | TACTGGTCTCCTCCAAAGAG |
|  | 239-2591(R) | 2591 | ACAGGCTGTCCTTCAATATGAGC |
|  | HXB2-3341(R)*^c^* | 3341 | GTAAATCTGACTTGCCCAATTC |
| RT | 239-2571 | 2571 | CTGCACCTCAATTCTCTCTTTG |
|  | 239-2675 | 2675 | GTAACAGGAATAGAGTTAGGTCCAC |
|  | 239-2786 | 2786 | ATTAAAGGGACAATCATGACAG |
|  | 239-2794 | 2794 | caatcatgacaggggacacc |
|  | 239-2841 | 2841 | TTTGCTAACAGCTCTGGGGATGTC |
|  | 239-4615(R) | 4615 | TCTGTCTGGCCACTATTCTG |
|  | 239-4751(R) | 4751 | TCCACTAGCTACATGTACTGCAAC |
|  | 239-4673(R) | 4673 | tttgcctgcccatgtatagcc |
|  | HXB2-3018 | 3018 | ACAGGGATGGAAAGGATCAC |
|  | HXB2-3145 | 3145 | TTAGAAATAGGGCAGCATAG |
|  | HXB2-3253(R) | 3253 | GTACTGTCCATTTATCAGGAT |
|  | HXB2-3509 | 3509 | CCAGTACATGGAGTGTATTATGAC |
|  | HXB2-3837 | 3837 | GTGAAATTATGGTACCAGTTAG |
| Tat | 239-4682 | 4682 | CAGAAAGGAGAGGCTATACATG |
|  | 239-5811 | 5811 | CTTAGCACTGAAAGTAGTAAGCG |
|  | 239-6474(R) | 6474 | AACTGGCAATGGTAGCAACACT |
| ENV | 239-6353 | 6353 | TCATTAGAATCCTCCAACGAGC |
|  | 239-6463 | 6463 | TCTAGAAGCATGCTATAACACATGC |
|  | 239-7016 | 7016 | CATCAACAACATCAACGACAGC |
|  | 239-7589 | 7589 | CAGTCACCATTATGTCTGGATTGGT |
|  | 239-7593 (R) | 7593 | CTATCATTGATTGGTTGTGAGTGG |
|  | 239-7697 (R) | 7697 | AGTATTGTTAGTTCCAGTATACCTGG |
|  | 239-8075 | 8075 | AGGTGGCAGAACTGTATCG |
|  | 239-8961 | 8961 | CTCTTGACTTGGCTATTCAGCAAC |
|  | 239-8969(R) | 8969 | TGGTATACTCTCGATAGCAAGG |
|  | 239-9064(R) | 9064 | CCACCCATATTGTAGGTAGGT |
|  | 239-9266(R) | 9266 | CTCCATGGAGTATTCATATACTG |
|  | 239-9703(R) | 9703 | GATCAAACTTCCATGCTAGAACC |
|  | 239-9735(R) | 9735 | CTAACATATGCCTCATAAGTGTAGG |
|  | 239-9878(R) | 9878 | AACATCCCCTTGTGGAAAGTC |

*^a^*Primers with "239-" prefix denote SIVmac239 primers based on the sequence of SIVmac239 (GenBank accession number M33262). Nucleotides of SIV are numbered beginning from the 5’-end of the SIV genome (i.e., position 1 of SIV corresponds to nucleotide 257 of the M33262 sequence).

*^b^*The "(R)" notation designates a reverse primer.

*^c^*Primers with "HXB2-" prefix denote HIV-1 HXBc2 primers (GenBank accession number K03455).
