# Supplementary material for: Variation of Human Immunodeficiency Virus Type-1 Reverse Transcriptase within the Simian Immunodeficiency Virus Genome of RT-SHIV
Source: PLoS One. 2014 Jan 31;9(1):e86997. doi: 10.1371/journal.pone.0086997 (PMC3909041; doi:10.1371/journal.pone.0086997)
Supplement: Table S2 — Primer Sequences for the Construction of site-directed RT mutants. (DOCX) [file pone.0086997.s002.docx]

**Table S2. Primer Sequences for the Construction of site-directed RT mutants.**

| Primer Name | Primer Sequence (5' → 3') |
| --- | --- |
| HXB2 – 2681 | TGTACAGAAATGGAAAAGGAA |
| 239-5294(R) | CTTCTGGGTACTACCTTAATGTC |
| K275R:mutF | TACCCAGGGATTA**G**AGTAAGGCA |
| K275R:mutR | TGCCTTACT**C**TAATCCCTGGGTA |
| G196R:mutF | ACTTAGAAATA**A**GGCAGCATAG |
| G196R:mutR | CTATGCTGCC**T**TATTTCTAAGTC |

The mismatching bases have been bolded and underlined.
