# Supplementary material for: Variation of Human Immunodeficiency Virus Type-1 Reverse Transcriptase within the Simian Immunodeficiency Virus Genome of RT-SHIV
Source: PLoS One. 2014 Jan 31;9(1):e86997. doi: 10.1371/journal.pone.0086997 (PMC3909041; doi:10.1371/journal.pone.0086997)
Supplement: Text S1 — 454 Inoculum Sequencing. (DOCX) [file pone.0086997.s003.docx]

**Text S1**

**454 Inoculum Sequencing**

**Methods**

### Isolation of viral RNA from the RT-SHIV innoculum

RT-SHIV RNA was isolated from 500 µL of cell-culture supernatant according to the RNA extraction component of the ultrasensitive virus load assay (UVLA) described in 2010 by Deere et al. [[1](#_ENREF_1)]. This procedure was originally adapted from the viral RNA isolation procedure described by Palmer et al. [[2](#_ENREF_2)] that is commonly used in the HIV-1 single copy assay (SCA). This protocol was modified from our previously published assay [[1](#_ENREF_1)] to include 7 units/sample of proteinase K from a buffered aqueous glycerol solution instead of 200 µg/sample of lyophilized powder. Extracted viral RNA was resuspended in 21.4 µL of nuclease free water.

**PCR amplification and 454 pyrosequencing**

Viral RNA was converted to cDNA using AccuScript High Fidelity Reverse Transcriptase (Agilent Technologies, Santa Clara, CA, USA). A 40 µL, random 9-mer primed cDNA synthesis reaction containing 20 µL of viral RNA template was prepared according to the manufacturer’s instructions. Reaction conditions were amended to use a 90 min 42°C elongation step. After cDNA synthesis, six regions of the RT-SHIV genome were separately targeted for PCR amplification and subsequent 454 pyrosequencing. Each region was approximately 450 bases in length and, when possible, overlapped by 20 bases. Specifically, we targeted two regions within RT spanning amino acids 41 to 296 (454-RT(1) and 454-RT(2) and four regions within envelope (Env) comprising the entirety of gp120 (454-Env(1),(2),(3),(4)). Primer sequences and amplicons are described in detail below. Amplicons were generated by the universal tag method [[3](#_ENREF_3)] using custom universal tag sequences TagF and TagR. All conventional PCR was performed using Phusion high-fidelity DNA polymerase (New England Biolabs (NEB), Ipswich, MA, USA) and a Mutltigene Gradient Thermal Cycler (Labnet, Woodbridge, NJ, USA). A table describing the reaction and thermal cycling conditions for conventional PCR procedures is provided below. First round 454 target amplification (454-Round 1) was performed for 25 cycles in a 50 µL final reaction volume containing 4 µL of cDNA template. Elongation times were approximately three times greater per kb than those recommended by the manufacturer. Addition of 454-titanium adaptor sequences and multiplex identifiers (MIDs) was performed in a second 20 cycle PCR reaction in a 30 µL final reaction volume (454-Round 2) using 0.5 µL of 454-R1 PCR product.

After electrophoresis through a 1.5% agarose gel, ethidium bromide stained amplicons were visualized using a Dark Reader transilluminator (Clare Chemical Dolores, CO, USA) and the correctly sized band was excised and purified using the NucleoSpin Gel and PCR Clean-up System (Macherey Nagel, Bethlehem, PA, USA). Quantitation was performed by fluorometry using the Quant-iT PicoGreen dsDNA Assay Kit (Invitrogen, Carlsbad, CA, USA). Amplicons were diluted in 5 mM Tris-HCl pH 8.0 to 2 x 10^8^ molecules/µL and then pooled in equal molar concentrations to create sequencing libraries. Emulsion PCR (emPCR) was performed using the titanium library-A emPCR reagents (Roche/454 Branford, CT, USA) according to the manufacture’s long-fragment reaction and cycling conditions that are described in technical bulletin No. 2011-011 “Amplicon Sequencing with Various emPCR Amplification Conditions”. Library titration analysis determined that an input ratio of 0.6 molecules per bead would result in an average enrichment of 5 to 10%. Bi-directional 454-pyrosequencing was performed using a GS Junior Sequencer (versions 2.5p1). Sequencing results were analyzed by the 454 GS Run Processor version 2.5p1 using the default amplicon signal processing pipeline.

**Mutation and statistical analysis**

The consensus sequence of the RT-SHIV innoculum was used to generate a reference sequence for all alignments in this study. Roche’s GS Amplicon Variant Analysis (AVA) software version 2.5p1 was used to de-multiplex, align, and detect putative mutations in standard flow-gram formatted 454-sequence data. For each amplicon, the differential abundance of specific mutations in complementary read orientations was compared. This analysis was used to remove the majority of mutations that were more than twice as abundant in one of the read orientations. Due to the high 454 sequencing error rate in homopolymer regions, insertion and deletion mutations were not considered. Finally, all substitution mutations greater than 0.5% of the population that met the previously described criteria were selected and further analyzed in Microsoft Office Excel 2007.

**Primer sequences**

| **Amplicon ID** | **Amino acids Amplified^a^** | **Primer ID** | **Primer Sequence 5’ – 3’** |
| --- | --- | --- | --- |
| Universal Amplicon Tags | | | |
|  | - | TagF | CGGAACTCACTGCTCATACC |
|  | - | TagR | CAGTCCAGCTACGCTGACTC |
| 454-Target Amplification | | | |
| 454-RT(1) | 41 to 175 | RT-454 (1) F | TagF-AAGCATTAGTAGAAATTTGTACAGAG |
|  |  | RT-454 (1) R | TagR-CATGTATTGATAGATAACTATGTCTGG |
| 454-RT(2) | 157 to 296 | RT-454 (2) F | TagF-ACAGGGATGGAAAGGATCAC |
|  |  | RT-454 (2) R | TagR-GCCAGTTCTAGCTCTGCTTCTTC |
| 454-Env(1) | -4 to 143 | Env-454 (1) F | TagF-GGCTAATACATCTTCTGCATC |
|  |  | Env-454 (1) R | TagR-CAAGAACTAGTCTCATTGACCA |
| 454-Env(2) | 135 to 273 | Env-454 (2) F | TagF-ACAACAACAGCATCAACAAC |
|  |  | Env-454 (2) R | TagR-CTAGTTCCATTAAAGCCAAACC |
| 454-Env(3) | 243 to 392 | Env-454 (3) F | TagF-CTCCAGGTTATGCTTTGCTTAGATG |
|  |  | Env-454 (3) R | TagR-GGAACTCTCCTCTGCAATTTGTC |
| 454-Env(4) | 383 to 526 | Env-454 (4) F | TagF-CAATTTGACGGCTCCTG |
|  |  | Env-454 (4) R | TagR-AGAACCCTAGCACAAAGACC |
| 454 – Barcoded Fusion Primers | | | |
|  | - | Universal F | Titanium(A)-MID#-TagF |
|  | - | Universal R | Titanium(B)-MID#-TagR |
|  | - | Titanium (A) | CGTATCGCCTCCCTCGCGCCATCAG |
|  | - | Titanium (B) | CTATGCGCCTTGCCAGCCCGCTCAG |
| 454- Multiplex Identifiers (MID) | | | |
| **MID 1** | ACGAGTGCGT | MID 10 | TCTCTATGCG |
| **MID 2** | ACGCTCGACA | MID 11 | TGATACGTCT |
| **MID 3** | AGACGCACTC | MID 13 | CATAGTAGTG |
| **MID 4** | AGCACTGTAG | MID 15 | ATACGACGTA |
| **MID 5** | ATCAGACACG | MID 16 | TCACGTACTA |
| **MID 6** | ATATCGCGAG | MID 19 | TGTACTACTC |
| **MID 8** | CTCGCGTGTC |  |  |

^a^For PCR primer pairs, the region shown depicts the amplified region after trimming PCR primers

**Reaction and cycling conditions for conventional PCR reactions**

| Reaction Conditions | | | | | | | | |
| --- | --- | --- | --- | --- | --- | --- | --- | --- |
| PCR Reaction Name | **Primers Used** | **Volume (µL)** | **Template (µL)** | **Primers [nM]^c^** | **Buffer (1X)** | **dNTP [mM]** | **Phusion^a^ (U/µL)** | **MgCl_2_^b^ [mM]** |
| **454-Round 1** | 454 - Target Amplification | 50 | 4 µL cDNA | 1000 | Phusion HF | 0.2 | 0.02 | 2.3 |
| **454-Round 2** | 454 - Barcoded Fusion Primers | 30 | 0.5 µL 454-R1 | 1000 | Phusion HF | 0.2 | 0.02 | 2.3 |
| Thermal Cycling Conditions | | | | | | | | |
| PCR Reaction | **Initial Denature** | **Cycles** | **Annealing** | **Elongation** | **Melt** | **Final Extension** | |  |
| **454-Round 1** | 98°C for 75 s | 25 | 61°C for 30 s | 72°C for 48 s | 98°C for 15 s | 72°C for 5 min | |  |
| **454-Round 2** | 98°C for 75 s | 20 | 64°C for 15 s | 72°C for 55 s | 98°C for 15 s | 72°C for 5 min | |  |

^a^ Phusion High-Fidelity DNA polymerase (New England Biolabs, Ipswich, MA, USA)

^b^ MgCl_2_ is present at 1.5 mM in 1X Phusion buffer. Reactions with >1.5 mM MgCl_2_ were supplemented with concentrated MgCl_2_.

^c^ Each primer is at the specified concentration

**References**

1. Deere JD, Higgins J, Cannavo E, Villalobos A, Adamson L, et al. (2010) Viral decay kinetics in the highly active antiretroviral therapy-treated rhesus macaque model of AIDS. PLoS One 5: e11640.

2. Palmer S, Wiegand AP, Maldarelli F, Bazmi H, Mican JM, et al. (2003) New real-time reverse transcriptase-initiated PCR assay with single-copy sensitivity for human immunodeficiency virus type 1 RNA in plasma. J Clin Microbiol 41: 4531-4536.

3. Daigle D SB, Pochart P. (2011) High-throughput sequencing of PCR products tagged with universal primers using 454 life sciences systems. Curr Protoc Mol Biol.
